# Compliance control based on PSO algorithm to improve the feeling during physical human–robot interaction

**DOI:** 10.1186/s40638-016-0052-0

**Published:** 2016-11-21

**Authors:** Zhongliang Jiang, Yu Sun, Peng Gao, Ying Hu, Jianwei Zhang

**Affiliations:** 1 Mechanical & Electrical Department, Harbin Institutes of Technology Shenzhen Graduate School, Shenzhen, China; 2 Shenzhen Institutes of Advanced Technology, Chinese Academy of Sciences, Shenzhen, China; 3 The Chinese University of Hong Kong, Hong Kong, China; 4 University of Hamburg, Hamburg, Germany

**Keywords:** Human–robot interaction, Surgical robot, Particle swarm optimization, Compliance control

## Abstract

Robots play more important roles in daily life and bring us a lot of convenience. But when people work with robots, there remain some significant differences in human–human interactions and human–robot interaction. It is our goal to make robots look even more human-like. We design a controller which can sense the force acting on any point of a robot and ensure the robot can move according to the force. First, a spring–mass–dashpot system was used to describe the physical model, and the second-order system is the kernel of the controller. Then, we can establish the state space equations of the system. In addition, the particle swarm optimization algorithm had been used to obtain the system parameters. In order to test the stability of system, the root-locus diagram had been shown in the paper. Ultimately, some experiments had been carried out on the robotic spinal surgery system, which is developed by our team, and the result shows that the new controller performs better during human–robot interaction.

## Background

Recently, more and more robots are brought to work with human. This is because human–robot cooperation can make full use of man’s wit to make up for robot’s poor intelligence, and we could complete work better. Human may keep in touch with a robot and engage in situations that people should exchange contact force each other when they work with robots. In addition, people may be required to keep in touch with robot, while they go to work together. Compared with the interaction between humans, the security of the human–robot interaction should be focused [1–3]. The collision should be detected quickly [4], and the robot needs to distinguish the intention which is unwished [5, 6]. When a physical force is exerted on the robot, the robot should respond quickly and steadily, just like force acting on someone’s arm, and we call this compliance control.

In the robotics community, there are a lot of robots that can complete the cooperative task with human. Furthermore, a large number of next-generation industrial robots emerge, which is lightly, compliant, and friendly [7]. The security of people, who work with robots, is one of the most important issues. This issue mainly depends on the detection of the collision. In other words, it depends on the perception of the force, and the time needed to response to the force. The collision detection system of robot [8] relies on a nonlinear adaptive impedance control law, while an image-based collision detector was used in [9]. In [10], a real-time filtering action on the currents of motors used to discriminate desired contacts and accidental collisions between human and robot. Intelligent robot is able to determine whether the input force is effective or not [11]. The most commonly used two compliant control methods are impedance control and force control [12, 13]. In addition, there have been many studies on the modeling of animal muscles [1, 11, 14]. We observe the muscle of cat’s legs when it lands and then establish the physical model of the single joint of robot by bionics knowledge. According to the physical model, the movement of RSSS II would be moved compliantly when it is pulled or dragged. In this controller, the actual current of motor is used as input variable. Then, the controller would output velocity of arm. Particularly, there is neither 6-Dof F/T sensor nor distributed tactile sensing on RSSS II. In order to realize the perception of the force, we only need to record the real-time current of the motor on each joint of the robot.

It is not easy to obtain the parameters of physical model directly (*J* moment of inertia; *B* viscous damping coefficient; *K* spring coefficient), so we intend to get the parameters through the PSO algorithm. We follow the method come up with by Gaing [14]. Firstly, the current and speed of the motors in joints should be recorded while the robot is pulled. Secondly, the values of parameters obtained by PSO algorithm would be closed to actual values. These parameters ensure the compliant of human–robot interaction. Finally, the stability can be tested by the root-locus method or the Routh criterion [15].

In fact, surgeons need to adjust the position of the robot’s arm during robot-assisted surgery [16]. This process is time-consuming and unsafe if it relies on remote control or preoperative planning. We obtain actual current of motor when one or multiple force acting at any position of the robot’s arm. Then, the speed of each joint can be calculated by controller. Finally, the robot’s arm can be moved to any desired position following the doctor’s hands.

In order to verify the compliance and stability of the control algorithm proposed in this paper, a number of experiments are performed on the RSSS II. The average rate of change, proposed to evaluate the compliance, is a new concept, which refers to the concept of smooth curve in math. Finally, it is proved that the controller proposed in this paper is superior to the proportional controller in compliance, and the stability of the algorithm is verified. The new controller can calculate the joint’s velocity instantly when there is a force acting on the robot.

 The rest of this paper is organized as follows. “Methods” section presents a control model and deduces the state space equation of the model. “Optimization of parameters” section obtains the closed-system parameters by PSO algorithm and tests the stability of controller, with the closed parameters, by root-locus method. “Experiments” section presents several experiments, and some conclusions can be known.

## Methods

### Biomimetic background

Muscles are usually considered as motors that produce mechanical work [17]. In fact, they perform multiple functions like brakes, dampers and struts [18]. For example, we observe the function of the muscles in animal’s leg, such as cat. First of all, joints remain flexible and muscles play a role of buffer at the moment of the cat landing. Muscles, which are similar to the action of the torsion spring, are the key organs to maintain stability.

### Physical and mathematical modeling

**Fig. 1:**
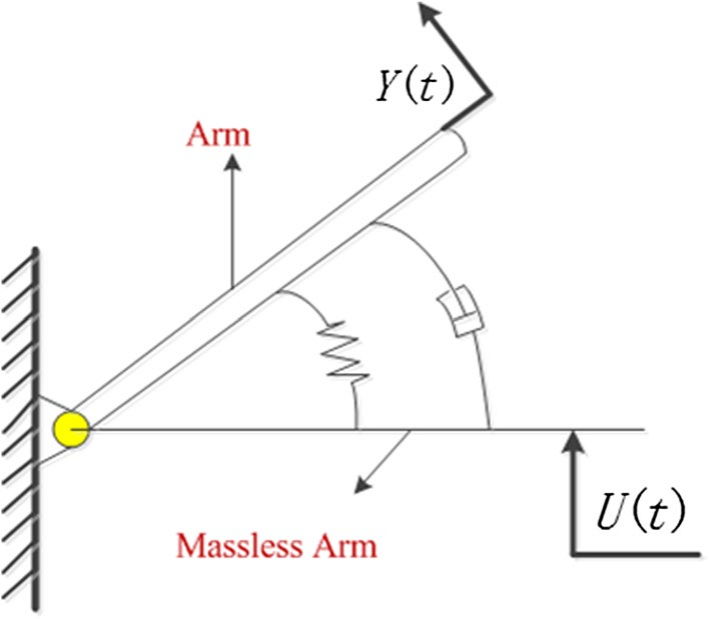
The spring–mass–dashpot system

Through the analysis of the process that a cat lands, the physical model of the single joint is established (in Fig. 1). A spring (torsional spring), which stores energy, is used as the muscles in cat’s leg. An elastic force is generated to hinder the movement along with the joint when the spring is stretched. In addition, elastic force is linear to deformation. The damper is used to consume energy to prevent the increase in the transient elastic force too much to cause damage, which is similar to passive muscle [19]. The positive direction of arm is according to the arrow in Fig. 1. When there is external force acting on the arm, the spring and the damper work together to promote the mass block move at a certain speed. Finally, the RSSS II moved to a new location which is the doctor required it being. So, doctors can adjust the arm of RSSS II easily during operation.

 The physical model of muscle in joint is shown in Fig. 1. In the model, the input of the system is *U* (*t*) which is the displacement of the massless arm. At *t* = 0, the massless arm is moved at a constant speed or in other words $\dot{U}$  = constant. The output is the displacement *y* (*t*) of the mass. (The displacement is relative to the initial position.) We assume that the friction force of the dashpot is proportional to $\dot{y} - \dot{u}$ and that the spring is a linear spring; that is, the spring force is proportional to *y* − *u*.

For the rotating system, the rotation law can be expressed as:

1 $$J \cdot \alpha = \sum T$$

where *J* is a moment of inertia, *α* is the acceleration of the object, and $\sum T$ is the sum of the moment acting on the object in the direction of the angular acceleration: *α*. The rotation law is applied to the system, and the inertia of the massless arm is zero.

2 $$J\frac{{{\text{d}}^{2} y}}{{{\text{d}}t^{2} }} = - B\left( {\frac{{{\text{d}}y}}{{{\text{d}}t}} - \frac{{{\text{d}}u}}{{{\text{d}}t}}} \right) - K\left( {y - u} \right)$$

or

3 $$J\frac{{{\text{d}}^{2} y}}{{{\text{d}}t^{2} }} + B\frac{{{\text{d}}y}}{{{\text{d}}t}} + Ky = B\frac{{{\text{d}}u}}{{{\text{d}}t}} + Ku$$

where *B* is viscous damping coefficient and *K* is spring coefficient.

### The state space equation building

Since integrators in a continuous-time control system serve as memory devices, the outputs of such integrators can be considered as the variables that define the internal state of the dynamic system. Thus, the outputs of integrators serve as state variables [17].

Next we shall obtain a state space model of this system. Then, we shall compare the differential equation for this system with the standard form:

4 $$\ddot y +a_1 \dot y+a_2 y=b_0 \ddot u +b_1 \dot u+b_2 u$$

We can obtain $a_{1}$, $a_{2}$, $b_{0}$, $b_{1}$, $b_{2}$, which represent the constant coefficients of the equation.

Define system state variables:

$$x_{1} = y - \beta_{0} u = y$$

5 $$x_{2} = \dot{x}_{1} - \beta_{1} u = \dot{x}_{1} - \frac{B}{J}u$$

where $$\beta_{0} = b_{0}$$, $$\beta_{1} = b_{1} - a_{1} \beta_{0}$$, $$\beta_{2} = b_{2} - a_{1} \beta_{1} - a_{2} \beta_{0}$$.

So state equation of the system can be obtained:

6 $$\left[ {\begin{array}{*{20}c} {\dot{x}_{1} } \\ {\dot{x}_{2} } \\ \end{array} } \right] = \left[ {\begin{array}{*{20}c} 0 & 1 \\ { - \frac{K}{J}} & { - \frac{B}{J}} \\ \end{array} } \right]\left[ {\begin{array}{*{20}c} {x_{1} } \\ {x_{2} } \\ \end{array} } \right] + \left[ {\begin{array}{*{20}c} {\frac{B}{J}} \\ {\frac{K}{J} - \left( {\frac{B}{J}} \right)^{2} } \\ \end{array} } \right]$$

and

7 $$\dot{y} = \dot{x}_{1} = \left[ {\begin{array}{*{20}c} 0 & 1 \\ \end{array} } \right]\left[ {\begin{array}{*{20}c} {x_{1} } \\ {x_{2} } \\ \end{array} } \right] + \frac{B}{J}u$$

where $${\mathbf{A}}\,({\mathbf{t}}) = \left[ {\begin{array}{*{20}c} 0 & 1 \\ { - \frac{K}{J}} & { - \frac{B}{J}} \\ \end{array} } \right]$$ is called the state matrix, $${\mathbf{B}}\,({\mathbf{t}}) = \left[ {\begin{array}{*{20}c} {\frac{B}{\text{J}}} \\ {\frac{\text{K}}{\text{J}} - \left( {\frac{\text{B}}{\text{J}}} \right)^{2} } \\ \end{array} } \right]$$ the input matrix, $${\mathbf{C}}\,({\mathbf{t}}) = \left[ {\begin{array}{*{20}c} 0 & 1 \\ \end{array} } \right]$$ the output matrix, and ${\mathbf{D}}\, ({\mathbf{t}} )= \frac{B}{J}$ the direct transmission matrix.

The state space equations of the system are given by (6), and the output equation is (7). (Note that this is just one of the numerous state space expressions for the given system.)

The direct transmission matrix **D** built direct mapping of the input and output. In other words, the system would respond to a given input signal if it is not zero. In addition, the presence of integrator would establish a connection between current state and future state. Moreover, integrator can prevent the output from being mutated.

## Optimization of parameters

PSO, firstly introduced by Kennedy and Eberhart [17], is one of the modern heuristic algorithms. The features of the algorithm are as follows.

### Design evaluation function

The reasonable evaluation function determines the speed of the optimization process, the convergence, and the rationality of the optimization results greatly. We defined the evaluation function given in (8) as the evaluation value of each particle in population.

8 $$F = \frac{1}{n}\mathop \sum \limits_{1}^{n} \left| {y - r} \right|$$

where *n* is the number of input; *y* is the output of the controller designed by the optimized parameters; *r* is the output of actual system. We deem the optimal parameters nice when the mean of difference between the actual output and the design output reached the minimum.

### The implementation of the PSO algorithm

**Fig. 2:**
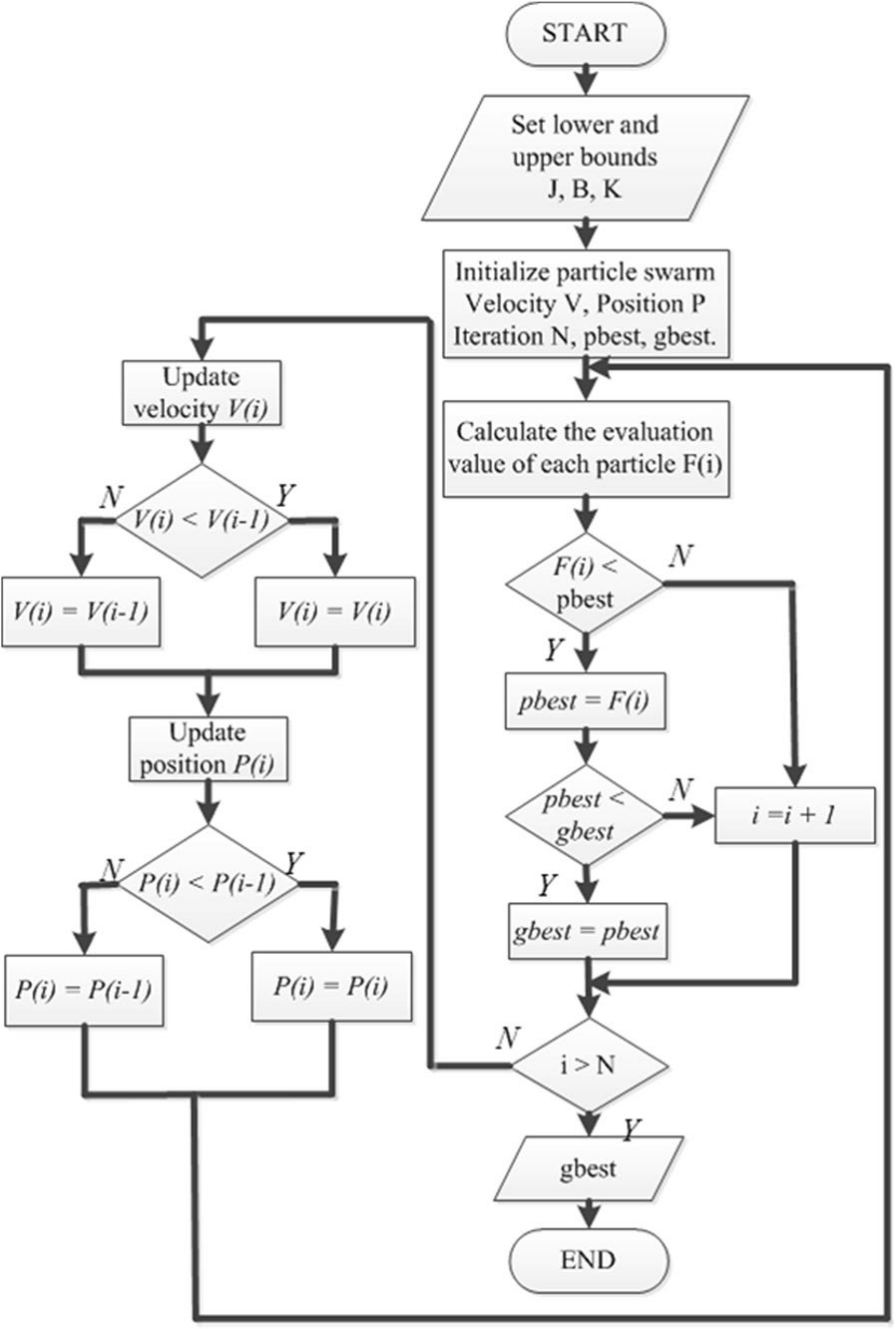
PSO algorithm

**Fig. 3:**
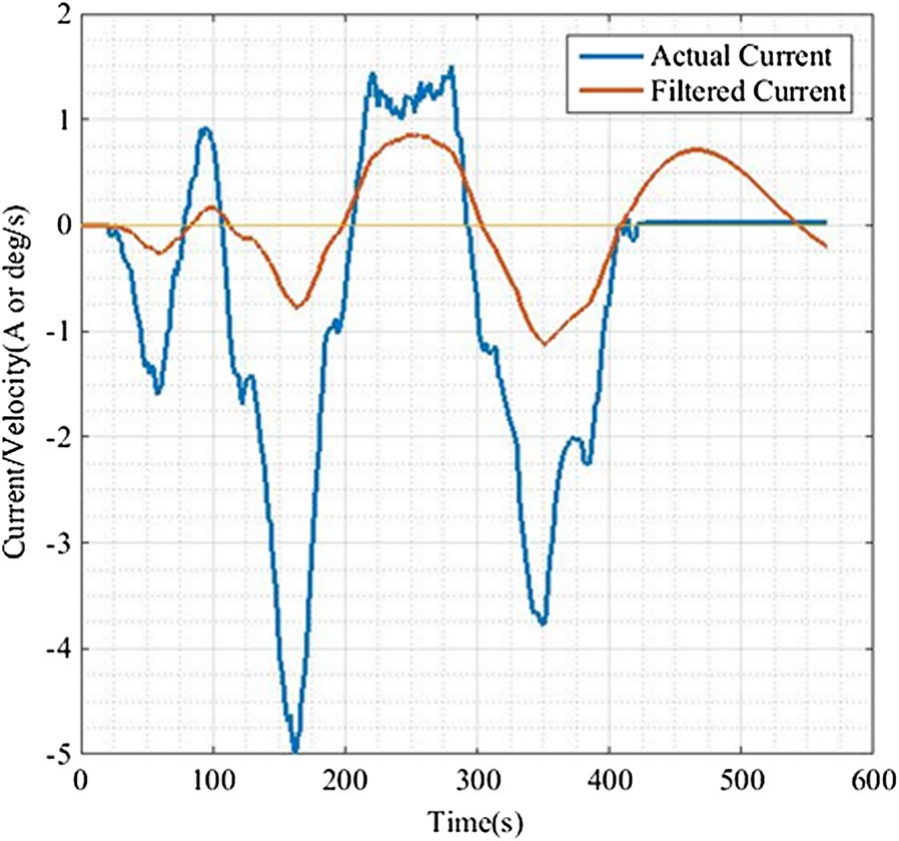
Actual current and filtered current

These parameters are intrinsic properties of a certain system. However, we cannot obtain the accurate values of system. Therefore, the PSO algorithm is used to obtain values which are close to the real system parameters (*J*, *B,* and *K*). The three parameters are composed of a three-dimensional particle **P** = [*J*, *B*, *K*]. Suppose that there are N particles in population, and the PSO algorithm was developed as in Fig. 2. The optimization result is **P** = [25.7337, 0.2906, 0.0149]. In order to obtain a smooth current as input signal, the mean filter was introduced in this paper (in Fig. 3).

### Verification of system stability

After obtaining the closed-system parameters, the stability of the system should be tested. The bad parameters may lead to unstable system. The most commonly used methods are root-locus method and the Nyquist curve among these methods. In this paper, the root-locus method is chosen because it is more convenient and intuitive.

In the state space, there are two methods to draw the root locus when the system equations are expressed in the state space. The first scheme is that we transformed the state space equations into a closed-loop transfer function and drawn the root locus according to the open-loop transfer function. The other approach is that we could draw a root locus directly by using the state space equation. We adopted direct method without extra calculation. The final result is shown in Fig. 2 from the system Eqs. (6) and (7).

**Fig. 4:**
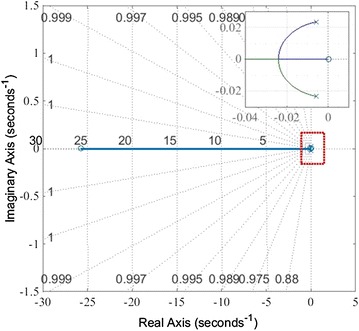
System root locus

The system is asymptotically stable because the characteristic roots of the closed-loop system are in negative real part. In Fig. 4, the poles of the closed-loop transfer function are all located in the left-half S plane. We can easily draw the conclusion that the system is stable.

## Results and discussion

We performed experiment, which uses the proposed controller, on RSSS II. RSSS II is serial-link robot with six degrees of freedom, and the end effector is a mechanism whose feed motion is independent; all joints are equipped with Maxon brushless DC motor and configured with high-resolution encoder; driver adopted COPLEY; principle computer adopted DELL mainframe; principle/slave computer established communication by CAN bus which is used widely.

In this paper, we verify the performance in compliance and stability of the control method, which is proposed in this paper, by comparing with proportional controller. We make robot seem human-like and make the human–robot interaction more convenient. This will improve the efficiency of the whole process of surgery and reduce the radiation of surgeon.

### Definition of a smooth curve in the field of calculus

 Function: $y = f(x)$, domain is $(t_{1} ,t_{2} )$,  if the derivative of the function exist and is continuous anywhere in $(t_{1} ,t_{2} )$, this curve can be regard as smooth curve.

In fact, there is no certain function expression and we only get the velocity at moment. We established the compliance index to evaluate the actual effect of control model by referring to the definition of smooth curve.

The first step: Obtain the absolute value of the difference between two adjacent points:

9 $$D\_val\,(n - 1) = \left| {y\,(n) - y\,(n - 1)} \right|$$

where *D_val* represents the absolute value of the difference between the adjacent points; *y* (*n*) indicates the value of the *N*th point; and *y* (*n* − 1) indicates the value of the *N* − 1th point.

The second step: Calculate the sum of *D_val*

10 $$val = \sum\limits_{1}^{n - 1} {D\_val}$$

The third step: Get the average change rate:

11 $$rate = \frac{val}{{(n - 1) \times {\text{inter}}}}$$

where rate is average rate of change; inter represents the time interval of the adjacent points, inter = 30 ms.

### Comparison of compliant

**Fig. 5:**
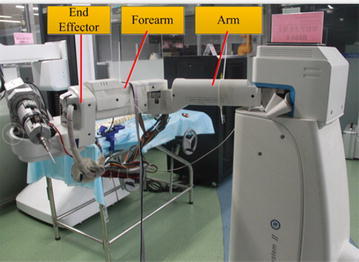
Structure of RSSS II

We make the force acting on the end effector, arm, and forearm, respectively, and change the direction of force constantly. Then, we observe the actual effect of the controller and export the real-time current value and speed value. In addition, the time interval of data collection is 15 ms. Details of RSSS II are shown in Fig. 5.

We conducted 30 experiments that we dragged and pulled the RSSS II, which are controlled by the method proposed in this paper, at end effector, forearm, and arm, respectively. We found that the values of every result of the average change rate are similar. A summary is included in Table 1, and the average change rate is calculated by (9)–(11).

**Table 1:** Spring–mass–dashpot system (SMD system) and P controller

Behavior	End effector	Arm	Forearm	Mean value
SMD system	0.0252	0.0257	0.0248	0.0252
P controller	0.0759	0.0583	0.0721	0.0688

The effective points of the end effector, arm, and forearm are 251, 664, and 564, respectively. Moreover, the evaluation method is correct logically because the variation of rate of change is less than 2%.

We design Table 2 in (12).

**Table 2:** Range of average rate

Behavior	End effector	Arm	Forearm
SMD system	0	1.9841	1.5873
P controller	10.3198	15.2616	4.7965

12 $$relat\_varition = \frac{{\left| {{\text{Rate}} - {\text{mean}}} \right|}}{\text{mean}} \times 100\%$$

**Fig. 6:**
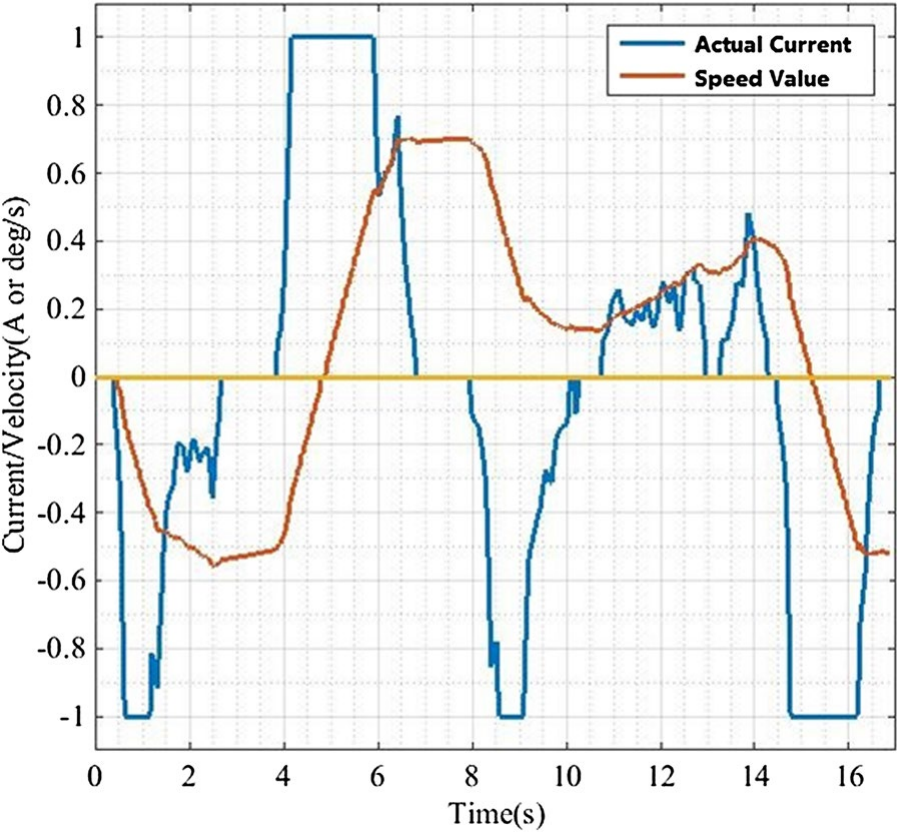
The *blue line* represents the actual control current, as the system input; *red line* is the corresponding speed values

The same signal is given as input to the new controller and proportional controller (the ratio of input current to output rate is 1:1). As shown in Fig. 6 and Table 1, we can know that the fluctuation of input current is larger. After processing the input current by the proportional controller, the result is not ideal due to the unstable motion of the robot. And the output curve of new controller is more smooth. It is better than proportional controller. The reason is that the new physical model is a second-order system with an integrator which has memory. In other words, the output of system is not only related to the current input, but also influenced by the present state. The average rate of change of the proportional controller is 2–5 times as much as that of the controller proposed in this paper in Table 2. Therefore, the feeling of the human–robot interaction is greatly enhanced and the compliance is promoted greatly.

### Stability of algorithm

**Fig. 7:**
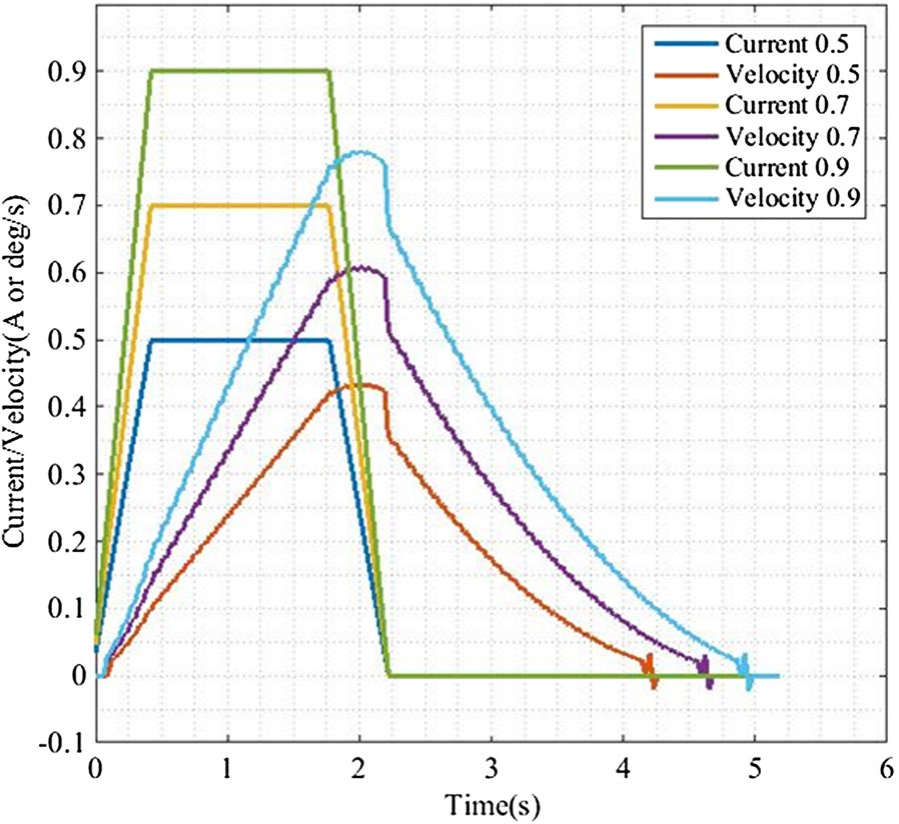
We set the initial state be 0. We displace variation of velocity when using difference step signals as input

Convergence should be the first to be considered when we evaluate a control system. We set the initial state to null. Then, we give some step-like signals whose values were 0.5, 0.7, and 0.9. The signals last for 1.8 s, and the variation of corresponding speed is shown in Fig. 7. It is clear that the output could converge to zero when the input has been zero no matter what input is.

On the basis of a large number of experiments, we found that the average change rate can only fluctuate within the 2% range if human applies force on the same position of RSSS II. The fact proved the feasibility and stability of the algorithm and the rationality of the evaluation criteria for the compliance of the system. In Fig. 6, it can be seen that we could get smooth velocity curve even if the input current fluctuated obviously. The robot can complete the following motion well, and the interaction force between human and robot is safe and reasonable in the whole process. On the other hand, we care for the convergence of the dynamic response process. In Fig. 7, the small fluctuations nearby the 0 point are due to the effect of the elastic elements in the model and the convergence is apparent. In summary, the compliance control is implemented.

## Conclusion

In this paper, physical model was built by referring the dynamic process of muscle in cat’s ankle joint. Then, the state space equation of the system was established which is not unique. The PSO algorithm was used to find the parameters which are close to real values of robot, and the stability of the system was verified by the root-locus method. Next, it could realize the following behavior when a force acts on the robot. In order to verify the stability and convergence of the controller, various pulses and square waves were used as input signal in experiments. The compliance has been defined as the evaluation index of human-like degree, and the average change rate was used to represent it. In the situation that the new controller and proportional controller took the same signal as input, the new controller possesses certain advantages by comparing different results. It performed better in physical human–robot interaction.

The method proposed in this paper made the physical human–robot interaction more human-like and realized coarse positioning during surgery. From the perspective of compliance and stability, the control method that is present in this paper is superior to traditional proportional controller. In addition, new control method does not need extra auxiliary equipment. Robot could realize the desired motion if it was pulled or dragged at any position. There is no force sensor in the whole process of the following behavior. These make the whole process of human–robot interaction more convenient. But, some problems have been found in the experiment; for example, there is zero drift which is irregular. The problem is caused by the arrangement of wire, which produces an additional traction force in the process during motion. In addition, we did not analyze the impact of convergence rate on the feeling of human which may be important in intention. Ultimately the virtual fixture would be applicated to improve the safety of RSSS II. I hope that these efforts would make the robot useful and improve the quality of robot-assisted surgery.
